# Unveiling immune-related gene signatures in triple negative breast cancer through integrated transcriptomic analysis

**DOI:** 10.37796/2211-8039.1708

**Published:** 2026-06-01

**Authors:** Priyanga Paranthaman, Ramanathan Karuppasamy, Shanthi Veerappapillai

**Affiliations:** Department of Biotechnology, School of Bio Sciences and Technology, Vellore Institute of Technology, Vellore, Tamil Nadu, India

**Keywords:** TNBC, Biomarkers, Protein, protein interaction, Module analysis, Tumor microenvironment, Drug, gene interaction analysis

## Abstract

**Background:**

Triple-negative breast cancer (TNBC) is an aggressive subtype with limited treatment options and poor prognosis. Understanding the underlying molecular mechanisms, particularly immune-related gene networks, is critical for identifying novel therapeutic targets.

**Aim:**

This study aimed to identify immune-related hub genes involved in TNBC progression by integrating microarray and RNA sequencing data.

**Methods:**

We integrated microarray and RNA sequencing datasets from five Gene Expression Omnibus (GEO) studies (GSE36295, GSE37751, GSE61724, GSE38959, and GSE58135) to identify differentially expressed genes (DEGs). Protein–protein interaction (PPI) networks were constructed using the STRING database. Key modules and hub genes were identified through network analysis. Functional enrichment was performed to elucidate biological pathways, while immune infiltration analysis assessed associations with the tumor microenvironment. Drug–gene interaction databases were queried for FDA-approved compounds targeting hub genes.

**Results:**

The PPI network revealed 179 nodes and 781 edges, indicating high connectivity. Module analysis highlighted a significant cluster with the identified key genes such as *CDK1, BUB1B, CCNA2, BUB1, CCNB1, KIF20A, CENPF, TOP2A, KIF11* and *MELK* validated at both mRNA and protein levels. Functional enrichment revealed pathways related to cell cycle control, chromosome segregation, and kinase activity. Immune infiltration analysis indicated involvement of B cells, macrophages, and neutrophils in the TNBC microenvironment. Drug–gene mapping revealed a lack of FDA-approved drugs targeting certain key hub genes.

**Conclusion:**

This integrative study identified key immune-related hub genes driving TNBC progression, with *KIF20A* emerging as a promising yet underexplored target. Drug repurposing strategies focusing on *KIF20A* and other identified hub genes may accelerate the development of effective treatments, offering valuable insights for future therapeutic and prognostic evaluations in TNBC.

## 1. Introduction

Triple-negative breast cancer (TNBC) is a highly aggressive subtype of breast cancer, characterized by the absence of estrogen, progesterone, and human epidermal growth factor receptor 2 (HER2). Conventional therapies such as chemotherapy, surgery and radiation frequently encounter constraints such as drug resistance, off-target toxicity, and possible recurrences. Due to the molecular complexity, TNBC necessitates comprehensive profiling to ascertain viable therapeutic strategies [1]. With advancements in next-generation sequencing (NGS) technology, biomarker identification can transform the treatment landscape of TNBC, enhancing diagnosis and patient stratification [2].

NGS technologies such as RNA-sequencing (RNA-seq) play a pivotal role in gene expression analysis [3]. RNA-seq maps sequencing data to determine gene expression without requiring prior genomic information. It offers several advantages, including isoform-specific measurements, a wider dynamic range, and the ability to detect single nucleotide changes [4]. Although RNA-seq is more costly, it complements traditional microarray technology due to its high reproducibility. The most common platform for microarrays is Affymetrix, which employs DNA spots on solid surfaces to measure gene expression and provides an affordable way to apply proven statistical techniques [5]. Various studies revealed the capability of these technologies to spot biomarkers for better diagnostic and treatment regimens. For instance, Liang et al. examined 91 genes associated with breast cancer in 156 individuals with inflammatory breast cancer (IBC) [6]. This research analyzed 51 TNBC patients by illumina sequencing, revealing increased somatic mutations in *TP53*, *BRCA1/2, NOTCH*, and DNA repair pathways relative to non-IBC. Lu et al. [7] spotted upregulated cyclins as possible biomarkers for TNBC using bioinformatic analysis of microarray and RNA-seq data. These cyclins were associated with decreased overall and progression-free survival. In addition, Li et al. [8] analyzed 405 TNBC cases and 128 normal tissue samples from 8 gene expression omnibus (GEO) datasets, identifying dysregulated pathways. A prognostic model comprising seven genes *(EXO1, SHCBP1, ABRACL, DMD, THRB, DCDC2, APOD)* was constructed, demonstrating robust predictive efficacy for TNBC prognosis. Meanwhile, Li et al. [9] investigated the tumor microenvironment (TME) with data from 158 TNBC patients in the TCGA-TNBC cohort. They discovered that lower immunogenic cell death (ICD) scores correlated with increased immune infiltration, tumor mutational burden, and immune pathway activity, underscoring their prognostic and therapeutic significance in immunotherapy. Xie et al. [10] highlighted the clinical utility of a 4-mRNA signature as a prognostic marker for disease-free survival, offering potential for guiding individualized treatment in breast cancer patients. In a more recent study, Krishnamoorthy et al. [11] combined microarray and RNA-seq data to identify hub genes, highlighting *AURKA, CCNB1, CDCA8, GAPDH*, and *TOP2A* as key players through protein–protein interaction (PPI) and module analysis.

Based on these studies the study aims to provide a comprehensive analysis of TNBC by leveraging advanced technologies to explore the molecular features of heterogeneous disease. Employing bio-informatics methodologies, we identify differentially expressed genes (DEGs) and construct PPI networks to elucidate essential biological determinants in the evolution of TNBC progression. The study also investigates gene functions, enriched pathways, immune cell infiltration, and potential Drug–gene interaction, offering new insights for developing targeted and personalized treatment strategies for TNBC.

## 2. Methods

### 2.1. Collection of transcriptomic datasets

Four microarray datasets (GSE36295, GSE37751, GSE61724, GSE38959) and one RNA-seq dataset (GSE58135) were downloaded from the publicly available GEO database and analyzed using the GEO2R script [12–17]. GEO datasets frequently lack therapeutic metadata such as specific drug regimens, doses, or treatment durations which may introduce bias in any downstream survival or prognostic analyses. We mitigated this by selecting only untreated baseline samples, thereby minimizing clinical variation. This study focused on the datasets that are fulfilling the following criteria: (i) presence of both control and TNBC samples, (ii) adequate sample size (≥4 per group) and (iii) no interventions with drugs or gene expression manipulation.

### 2.2. Data preprocessing and identification of DEGs

The microarray and RNA-seq data were processed using the limma and DESeq2 R packages, respectively, for normalization, log2 transformation and DEG screening. For microarray data, the expression matrix was retrieved using the GEO-query package, and log2 transformation was applied based on intensity quantiles. Samples were grouped, and a linear model was fitted to the data. The statistical significance (p-value) for each DEG was then determined using the empirical Bayes model. For RNA-seq, raw gene-level counts were processed using the DESeq2 package. Genes with low expression (fewer than 10 counts in the smallest group) were filtered out. A DESeqDataSet was constructed with group information (TNBC vs Control), and normalization was performed using DESeq2’s median-of-ratios method. Differential expression was assessed using the Wald test. The p-values were corrected using the Benjamini– Hochberg method to control the false discovery rate (FDR). Genes with FDR <0.05 and log2 fold change (FC) ≥ ±1.0 were identified as DEGs, and the overall pattern of gene expression in the dataset was visualized using a volcano plot by employing the ggplot2 R package. Additionally, to analyze the intersecting upregulated and downregulated genes, the web-based tool InteractiVenn was utilized [18].

### 2.3. Protein–protein interaction network construction

The DEGs are functionally essential and highly interconnected with other genes, thus analyzing their PPI network shed light on the mechanisms between genes and their biological modules [19]. The STRING plugin in the Cytoscape software (v3.9) was used to illustrate the PPI network of the statistically significant DEG with a combined confidence score of ≥0.9. The highly clustered connections were identified using Molecular Complex Detection (MCODE) parameters such as K-Core = 2, degree cutoff = 2, and maximum depth = 100. Additionally, the topological parameters in the cytoHubba including MNC, MCC, Degree, Stress, Radiality, Betweenness and Closeness were utilized to detect the hub genes.

### 2.4. Functional enrichment analysis

A comprehensive analysis of gene ontology (GO) and Kyoto Encyclopedia of Genes and Genomes (KEGG) pathway enrichment was conducted for the identified hub genes using the ShinyGO (v0.77) tool. The GO analysis encompassed three distinct modules such as biological process (BP), cellular component (CC), and molecular function (MF). The molecular interactions among hub genes were depicted graphically, considering a significant FDR <0.05.

### 2.5. Cross-validation of hub gene mRNA and protein level expression

To validate the expression levels of hub genes, GEPIA2 tool was employed to analyze the expression of key genes in BRCA tissues compared with normal tissues from TCGA and GTEx data [20]. Further, the protein level expression of hub genes in the breast tissue was predicted using the online tool, Human Protein Atlas (HPA) [21]. The staining intensity (strong, moderate or weak) and the percentage of stained cells (>75%, 25–75%, or < 25%) were used to classify the expression levels in HPA into four groups: high, medium, low, and not detectable.

### 2.6. Prognosis analysis of the hub genes

The Kaplan-Meier Plotter was utilized to investigate the predictive impact of hub genes in breast cancer [22]. The instances in the database were separated into two categories based on the mRNA expression of the genes, with the top 50% belonging to the high expression group and the lowest 50% belonging to the low expression group. The hazard ratio (HR) and log rank P value for each hub gene were calculated.

### 2.7. Immune infiltration, regulatory network and drug–gene interaction analysis

The TIMER2.0 server was employed to examine the correlation between hub gene expression and immune infiltrates in the BRCA dataset within the TCGA cohort [23]. Specifically, immune infiltration estimations using the TIMER2.0 web server integrates multiple state-of-the-art algorithms such as TIMER, xCell, MCP-counter, CIBERSORT, EPIC, and quanTIseq via the immunedeconv R package. These tools have been benchmarked for robustness across different tissue types and immune cells. The gene expression profiles from TCGA cohorts were used as input, and the resulting immune cell-type associations were analyzed to identify relevant immune signature-related genes. Notably, TIMER is the only algorithm that explicitly accounts for tissue specificity when estimating immune cell populations. Thus, the Gene module in immunological association was utilized to examine the relationships between hub gene expression and several tumor-associated immune cells, including B cells, CD8^+^ T cells, CD4^+^ T cells, macrophages, neutrophils, and dendritic cells (DCs). To account for the impact of tumor purity on immune infiltration levels in clinical samples, we adjusted the purity of the relevant analysis. The p-value <0.05 was considered statistically significant to identify the prominent correlation between the hub genes and immune cells for this analysis.

**Fig. 1:**
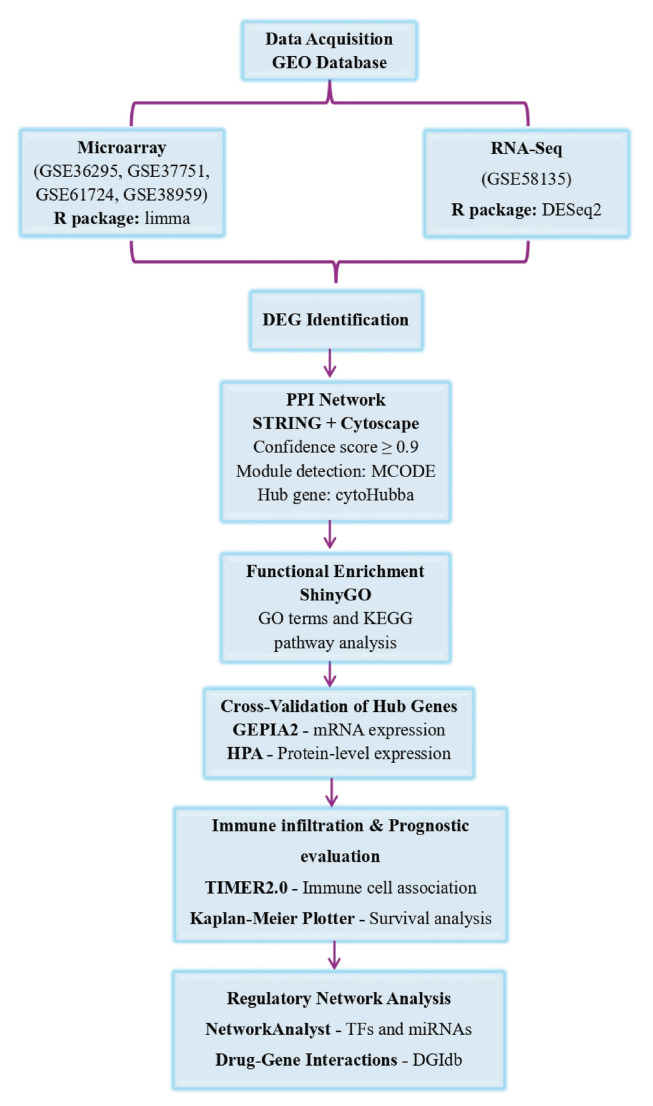
Overall schematic representation of the study design and analytical workflow.

The transcriptional and post-transcriptional regulatory elements, including transcription factors (TFs) and microRNAs (miRNAs), were systematically examined for the identified hub genes. The interaction networks were constructed using NetworkAnalyst [24]. The JASPAR database was used to uncover the TFs, which consists of curated and non-redundant experimentally validated TF binding sites [25]. miRNAs were determined through the miRTarBase v8.0 database, which comprises experimentally validated miRNA target genes from several species [26]. In addition, the potential key genes were examined for their prospective drugs by utilizing the Drug–gene interaction database (DGIdb v.5.0.3) [27]. DGIdb compiles information from various sources including DrugBank, ChEMBL, NCBI Entrez, Ensembl, OncoKB, Pub-Chem, clinical trials, and PubMed literature, to predict possible clinical therapeutic drugs associated with the hub genes. Finally, Cytoscape was employed to build the network of Drug–gene interaction [28]. The overall schematic representation of the study design and analytical workflow is depicted in Fig. 1.

## 3. Results

### 3.1. Identification of DEGs

**Fig. 2:**
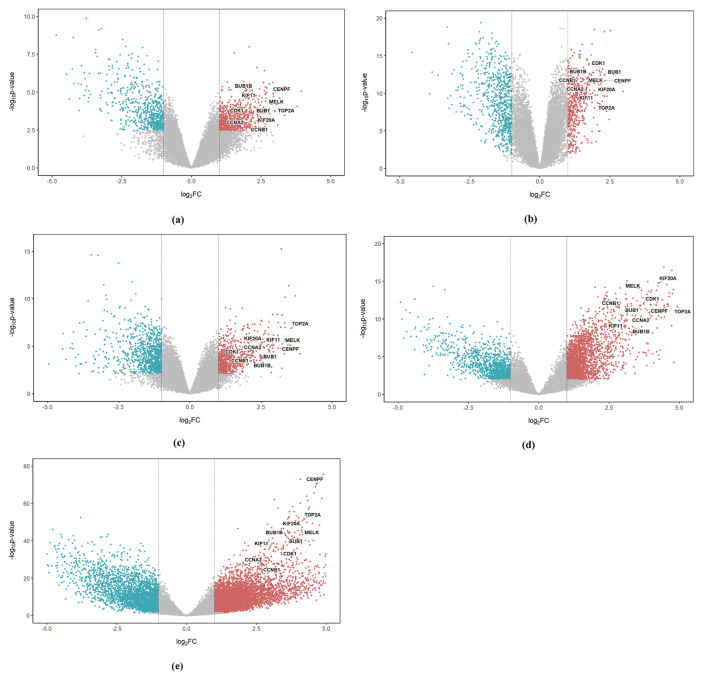
Volcano plot of differentially expressed genes on analysing (**a**) GSE36295 (**b**) GSE37751 (**c**) GSE61724 (**d**) GSE38959 (**e**) GSE58135 datasets. Red and blue dots represent up-regulated and downregulated genes respectively. Grey dots represent insignificant genes.

**Fig. 3:**
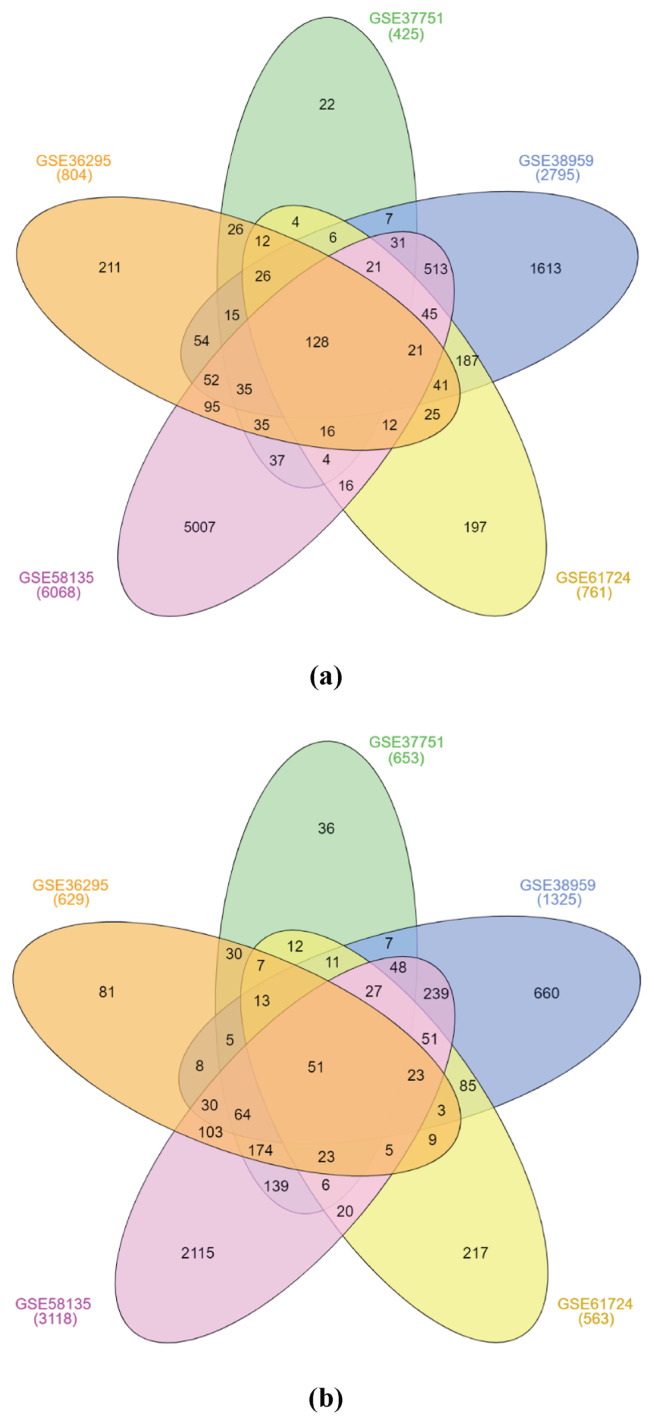
The overlap of (**a**) upregulated and (**b**) downregulated differentially expressed genes identified across five GEO datasets.

Identifying key coding genes in cancer tissues is essential for designing effective therapeutic agents in pharmacological research. On screening the DEGs according to the criteria, 1,433, 1,078, 1,324, 4,120, and 9186 genes were identified from GSE36295, GSE37751, GSE61724, GSE38959, and GSE58135, respectively. The detailed information for the GEO datasets is shown in Table 1. The distribution of the DEGs is typically visualized using a volcano plot. Each point on the plot in Fig. 2 represents a gene and helps to simultaneously assess the statistical significance and magnitude of gene expression changes. We further identified 179 overlapping DEGs among these five datasets, of which, there were 128 upregulated and 51 down-regulated genes (Fig. 3).

### 3.2. Network construction and significant module analysis

**Fig. 4:**
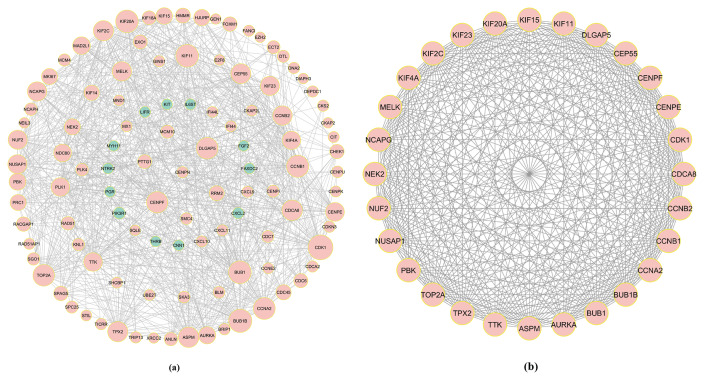
Protein–protein interaction network and cluster identification using cytoscape (**a**) Interaction network of up-regulated (Pink) and down-regulated (Green) genes (**b**) Highly interconnected subnetwork identified using MCODE plugin.

The STRING server analysis of common DEGs yielded a network comprising of 179 nodes and 781 edges. The average node degree was found to be 8.73, with an average clustering coefficient of 0.47. Additionally, the PPI enrichment p-value was found to be less than 1.0e-16, indicating that the proteins are at least partially biologically connected as a group. The strong relationship between the genes, as specified by a combined score of more than 0.9, was visualized in Cytoscape (Fig. 4a). The network visualization provided an overview of gene relationships, and further analysis with the cyto-Hubba revealed the topological importance and centrality measures of individual genes within the network (Table 2). Subsequently, the MCODE plug-in results disclosed a total of 8 clusters, of which the module with the highest score of 25.481 (24 nodes and 344 edges) was extracted separately according to the connective degrees and visualized (Fig. 4b). Interestingly, the top 10 common genes identified by the seven algorithms from cytoHubba entirely overlapped within the highest score module from the MCODE. These findings lay a solid basis for further functional validation and in-depth studies to elucidate their specific roles and mechanisms.

### 3.3. Functional enrichment analysis

**Fig. 5:**
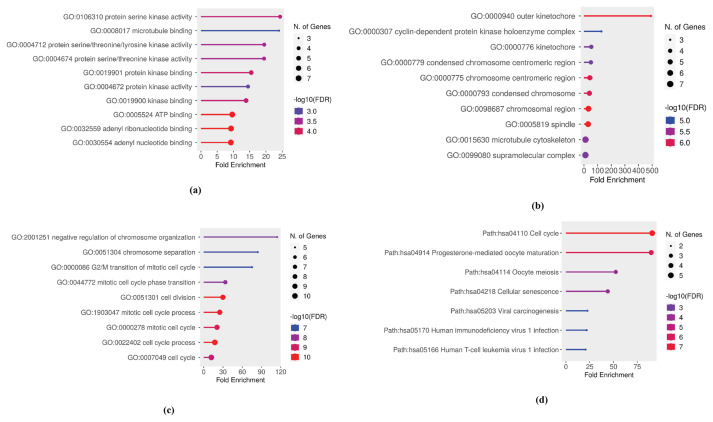
Functional enrichment analysis of hub genes (**a**) Molecular functions (**b**) Cellular components (**c**) Biological process and (**d**) KEGG pathways.

Functional enrichment analysis for hub genes offers a valuable insight into their functions and potential contributions to disease. The functional enrichment analysis of the 10 hub genes unveiled their involvement in crucial molecular functions, notably encompassing kinase-related activities (e.g., protein serine/threonine/tyrosine kinase), nucleotide binding, and microtubule interactions, as depicted in Fig. 5a. Analysis of cellular components indicated a significant concentration of common genes in pivotal roles such as kinetochore, centromeric regions, spindle, and microtubule cytoskeleton in chromosome segregation during cell division (Fig. 5b). Moreover, the biological processes primarily center on cell cycle-related processes, particularly those governing mitosis and chromosome organization (Fig. 5c). In the subsequent KEGG analysis, the hub genes were found to be highly enriched in pathways characterized by complex interplay between cell cycle regulation, reproductive pathways, and viral infections (Fig. 5d). The statistical significance of these enrichments is confirmed by the -log10(FDR) values, which indicate the probability of observing these gene overlaps by random chance. The higher the -log10(FDR) value, the more statistically significant the enrichment. In addition, higher fold enrichment indicates that genes in a specific pathway are significantly over-represented compared to chance, implying a stronger biological association.

### 3.4. Validation of mRNA and protein level expression of overlapped hub genes

**Fig. 6:**
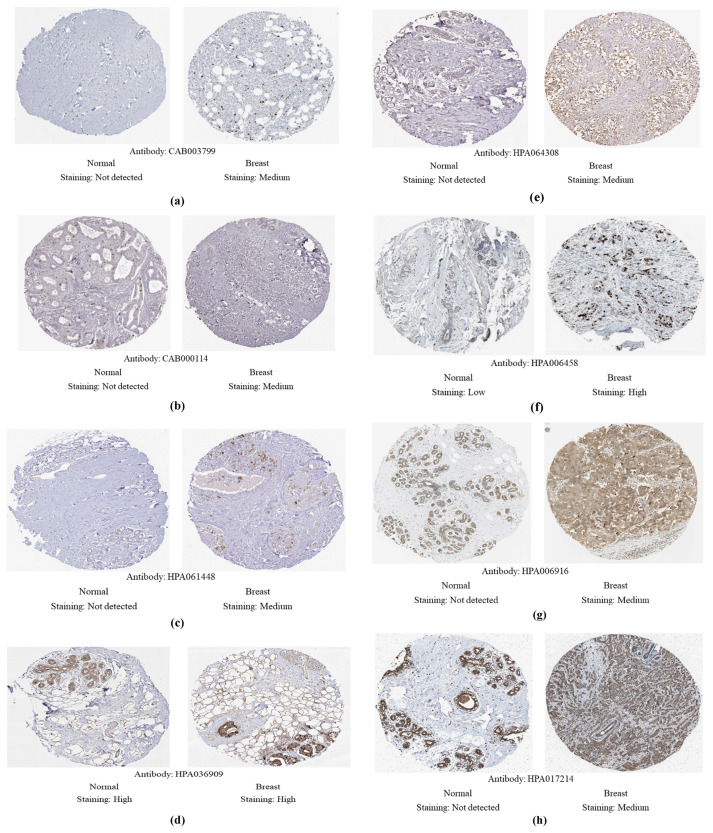
Immunohistochemistry of normal and tumor breast tissues from HPA database. (**a**) CDK1 (**b**) CCNA2 (**c**) CCNB1 (**d**) KIF20A (**e**) CENPF (**f**) TOP2A (**g**) KIF11 (**h**) MELK.

Them RNA expression levels of the hub genes were analyzed using the GEPIA box plot with 1085 breast tumor tissues and 291 normal tissues (Fig. S1a–j (https://www.biomedicinej.com/cgi/editor.cgi?article=1708&window=additional_files&context=biomedicine)). For the protein level expression, *CDK1, CCNA2, CCNB1, CENPF, KIF11 and MELK* were not detected in normal tissues, while medium expression levels of these genes were observed in breast tissues (Fig. 6a–c, e, g and h). Conversely, *KIF20A* showed higher protein expression in both normal and cancerous tissues, suggesting a complex regulation that warrants further investigation (Fig. 6d). Additionally, the low-level protein expressions of *TOP2A* were observed in normal tissues, whereas it is high in breast tissues (Fig. 6f). Overall, the transcriptional and translational expression levels of the hub genes emphasizing the potential of the identified hub genes as therapeutic targets.

### 3.5. Immune cell infiltration and prognostic value of the hub genes

To further understand the relationship between hub genes and immune cell infiltrations, we used TIMER2.0 to explore their association (Fig. S2a–j (https://www.biomedicinej.com/cgi/editor.cgi?article=1708&window=additional_files&context=biomedicine)). As detailed in Table 3, a statistically significant correlation was observed with the tumor’s purity (the proportion of actual cancer cells), and the presence of various immune cells within the tumor. Further, the relationship between elevated expression of hub genes and overall survival (OS) in BC patients was analyzed by the Kaplan-Meier survival analysis across the TCGA BC cohorts to study the prognostic value of each hub gene. The findings are shown in Fig. S3a–j (https://www.biomedicinej.com/cgi/editor.cgi?article=1708&window=additional_files&context=biomedicine).

### 3.6. Regulatory network and drug interaction analysis

**Fig. 7:**
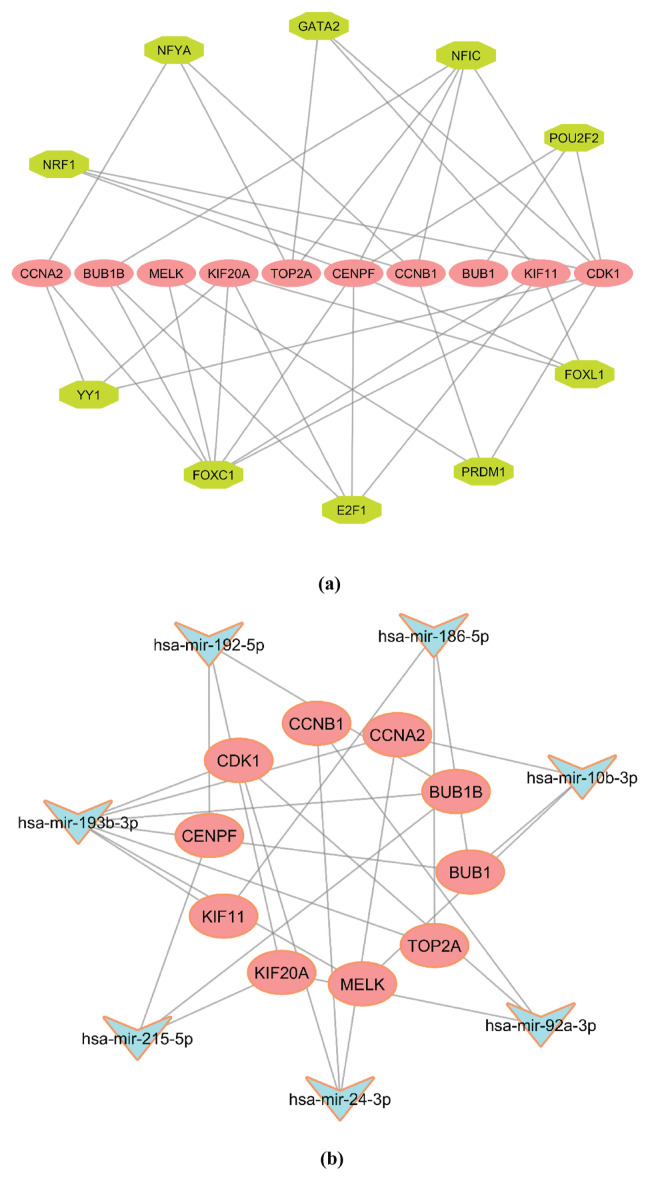
Regulatory network analysis (**a**) Transcription factor (green) - hub gene (pink) and (**b**) miRNA (blue) - hub gene (pink) interactions.

**Fig. 8:**
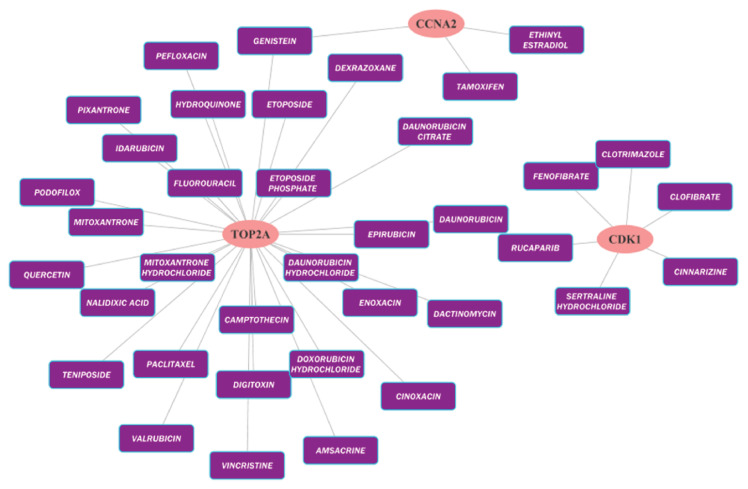
Protein drug interaction network of hub genes. The violet rectangles denote approved drug molecules, while the pink ellipse represents hub genes.

A regulatory cascade essential for appropriate execution of several biological events is triggered through a combinatorial action of miRNAs and TFs [29]. Thus, in the present study regulatory network analysis of TF and miRNA hub gene interactions were studied. Fig. 7a and b represents the TFs and miRNAs regulating the identified hub genes. Using DGIdb, we identified 38 FDA-approved drugs that specifically target hub genes, making them promising candidates for therapeutic applications and drug development (Fig. 8). Based on the interaction scores, the top 15 drugs (ranging from 0.157 to 1.053) are listed in Table 4.

## 4. Discussion

Immunotherapy emerges as a promising treatment strategy for TNBC, leveraging its high tumor mutation burden and tumor-infiltrating lymphocytes (TILs). Transcriptomic profiling, especially next-generation sequencing, offers a comprehensive view of TNBC, identifying biomarkers and gene signatures for personalized treatment and targeted therapies. Our study implemented a novel strategy by integrating microarray and RNA seq data to uncover crucial genes driving TNBC progression. To the best of our knowledge, this study integrates five datasets, providing a comprehensive view of the key genes involved in its progression. The datasets, sourced from patient-derived breast tissues (microarray) and primary tumors or cell lines (RNA-seq), focus on TNBC in comparison to normal tissue or other breast cancer subtypes. They span diverse populations across different geographic and demographic backgrounds, including both fresh-frozen and formalin-fixed paraffin-embedded (FFPE) samples, and feature two major gene expression platforms such as Affymetrix/Agilent microarrays and Illumina HiSeq. Together, they provide a rich molecular insight into the transcriptional diversity and clinical variability of TNBC, with data from patients in Saudi Arabia to the U.S. cohorts. Through network analysis, we identified 10 hub genes, including *CDK1, BUB1B, CCNA2, BUB1, CCNB1, KIF20A, CENPF, TOP2A, KIF11 and MELK.* Several of the identified hub genes were reported to be overexpressed in the recent literature evidence which in turn highlights the credibility of our analysis. For instance, the cyclin, *CDK1* is a key regulator of cell cycle progression, driving the transition from the S or G2 phase into mitosis. The accumulation of cyclins at various cell cycle stages is regulated by transcription and decreased protein degradation, allowing for proper cell cycle entry and progression [30]. Recently, it has been reported that *CDK1* is overexpressed in TNBC, reinforcing its potential as a therapeutic target [31]. Similarly, *CCNA2*, which involves in the transitions of G1/S and G2/M, has been identified as a prognostic marker for BRCA patients and are linked to tamoxifen resistance [32]. *CCNB1*, which is primarily active during the G2/M phase of the cell cycle, plays a crucial role in the regulation of mitosis and functions as a prognostic biomarker for BRCA survival and treatment resistance. The findings by Lu et al. [7] highlighted that *CDK1, CCNB1*, and *CCNA2* are significantly overexpressed in TNBC associated with aggressive tumor characteristics. *BUB* gene family is a highly conserved serine/threonine kinase that plays a crucial role in spindle checkpoint regulation. *BUB1* is required for spindle assembly checkpoint signaling and proper chromosome alignment, while *BUB1B*, inhibits late anaphase-promoting complex/cyclosome activity by regulating *CENPE*-dependent kinetochore function [33]. *BUB* genes are frequently upregulated in solid malignancies, including breast cancer, where its overexpression is linked to poor outcomes, especially in TNBC [34]. *KIFs*, kinesin superfamily proteins are microtubule-dependent molecular motors that play a vital role in mitosis, meiosis, and complex biological processes like learning, memory, and left–right asymmetry development. *KIF20A*, a member of the kinesin protein family, helps in chromosome transport during mitosis and is vital for proper cell division. Yang et al. [35] found that *KIF20A* may contribute to treatment resistance by modifying the TME in BC and works as a predictive and therapeutic response marker. On the other hand, *KIF11*, essential for spindle integrity and chromosome segregation was reported to be overexpressed in TNBC cell lines and is associated with poor prognosis [36]. *TOP2A*, a DNA topoisomerase enzyme, is crucial for gene transcription and cellular replication. *TOP2A* expression is abnormal in a variety of cancer types, including breast cancer, and it contributes to tumor growth and treatment resistance. Notably, in TNBC, high *TOP2A* expression has been linked to increased cell proliferation and invasiveness [37]. Clinical investigations indicate that patients with TNBC displaying aggressive characteristics often demonstrate *TOP2A* amplification. This correlation underscores the potential utility of *TOP2A* as a biomarker for disease severity and a possible therapeutic target for improving TNBC treatment strategies [38]. *CENPF* is an essential component of the cell cycle regulatory machinery and plays a key role in the kinetochore complex formation. Emerging research suggests that increased *CENPF* expression is associated with several aggressive malignancies, such as prostate, breast, and nasopharyngeal carcinoma, where it promotes tumor growth and metastasis. It is also involved in chemotherapy mediated resistance in TNBC, highlighting its potential as a novel target to enhance treatment efficacy [39]. *MELK* serves as a critical regulator of intercellular signaling, influencing a variety of biological and cellular mechanisms. Increased expression of *MELK* is commonly observed in breast cancer and is significantly associated with unfavorable clinical outcomes [40]. Li et al. [41] reported that the oncogenic role is associated with its ability to regulate different phases of the cell cycle through distinct mediators, where its suppression induces apoptosis in MDA-MB-231 cell lines. The GO terms and KEGG pathway analysis also demonstrated that the hub genes were enriched in the stemness of tumor cells and various cancer progression activities.

To determine the expression levels of hub genes at the mRNA and protein levels, the study examined tissue samples of breast cancer patients with the healthy controls. The mRNA expression level is significantly higher in tumor tissues than in normal tissues, confirming the upregulation of hub genes in breast cancer samples (p-value <0.05). Subsequently, we assessed the relative position and abundance of proteins using an immunohistochemistry (IHC) based protein expression pattern. Notably, the protein-level expression data for *BUB1B* and *BUB1* were unavailable, and we speculate that these genes might also be associated with breast cancer, but further research is required to definitively establish this relationship. The association between the hub genes and immune cell infiltration was subsequently analyzed. The purity exhibited a positive correlation with the expression of all hub genes (correlation coefficient (Rho), ranging from 0.130 to 0.246; p < 0.05). This finding suggests that these hub genes are predominantly expressed in tumor cells and act as indicators of tumor aggressiveness. In addition, we observed that these hub genes presented significant associations with infiltrating levels of B cells, T cells, macrophages, neutrophils, and DCs. For instance, the genes *CDK1, BUB1B, BUB1, CCNB1, KIF20A*, and *MELK* showed the strongest correlation with B cells (Rho, 0.069 to 0.100; p < 0.05). For macrophages, the genes *CCNA2, TOP2A*, and *MELK* were most significantly correlated with Rho value of −0.075 to 0.186 (p < 0.05). In case of DCs, the genes *CCNA2, BUB1, KIF20A, CENPF*, and *MELK* showed the positive correlation with Rho value of 0.067–0.129 (p < 0.05). This finding indicates that these hub genes were all significantly associated with tumor-associated B cells, macrophages and DCs in the BC microenvironment. It is worth mentioning that all the hub genes were positively correlated with neutrophils with Rho values ranging from 0.235 to 0.401 (p < 0.05). Emerging evidence highlights that neutrophil serve as a significant prognostic marker due to their pro-tumorigenic role in TNBC, particularly through neutrophil extracellular traps and pre-metastatic niche formation [42]. The remaining immune cells, CD4^+^ and CD8^+^ T cells, showed weak correlations with the hub genes, which were not statistically significant, as indicated by p-values greater than 0.05. Fu et al. [43] showed that a 25-gene immune-centric signature, enriched for B and T cell receptor pathways, predicted pathological complete response (pCR) to paclitaxel-and-anthracycline-based neoadjuvant chemotherapy more accurately than conventional clinical markers such as ER, PR, HER2, and Ki-67 status. Similarly, Lu et al. [44] developed the ImPredict score using gene expression data and machine learning in patients with HER2-negative breast cancer. This model successfully predicted CR to chemoimmunotherapy and outperformed PD-L1 testing in identifying patients most likely to benefit from immune checkpoint inhibitors [44]. Collectively, the infiltration results highlight the crucial role of the hub genes in modulating the tumor microenvironment of breast cancer, potentially impacting the development and progression of the disease. In the survival analysis, the HR and Logrank P values are the statistical measures indicating the strength of the association between hub gene expression and survival. Higher HR indicates the higher risk of mortality. Patients with BC who have high expression levels of *BUB1B, CCNA2, BUB1, CCNB1, KIF20A, CENPF* and *MELK* are predicted to have a worse OS than those with low levels. Similarly, the lower expression of *TOP2A* and *KIF11* genes were significantly correlated with worse prognosis and lower OS in BC patients. However, no significant difference in OS between low and high expressing cells of *CDK1* were noted. This suggested that abnormal expression levels of these hub genes at diagnosis can be considered as unfavourable prognostic genes, which can shorten the OS of BC patients.

Regulatory mechanisms frequently entail numerous TF binding collectively to the DNA as a unified complex. However, only a small portion of the regulatory partners for each TF is yet identified. This study uncovered 10 transcription factors linked to hub genes with a degree of 2. Among these, the members of the *GATA* family are pivotal TFs that regulate various pathways. In breast cancer, *GATA2* has been identified as a significant epigenetic regulator of G9a, influencing tumor survival and progression [45]. *NFIC*, a nuclear factor I (NFI) family member, functions as a TF in numerous physiological and pathological processes, including cancer. Particularly, the *NFIC1* TF serves as a tumor suppressor in TNBC by inhibiting MDA-MB-231 cell migration and invasion through S100A2-mediated suppression of the *MEK/ERK* pathway [46]. POU Class 2 Homeobox 2 (*POU2F2*) gene encodes a TF that activates PTPRG-AS1, modulating ferroptosis and proliferation via the miR-376c-3p/*SLC7A11* axis, thereby promoting TNBC progression [47]. The Forkhead Box (*FOX* ) family of TFs contains two important members involved in the development of TNBC, *FOXL1* and *FOXC1*. *FOXL1* overexpression in MDA-MB-231 cells decreases β-catenin, c-Myc, and cyclin D1 levels, hinders proliferation, invasion, and migration *in vitro*, and slows tumor growth *in vivo* by inactivating the Wnt/β-catenin signaling pathway [48]. *FOXC1*, on the other hand is consistently expressed in basal-like breast cancer, such as triple-negative tumors. *FOXC1* increases tumor aggressiveness via stimulating NF-κB signaling, epithelial-to-mesenchymal transition (EMT), improving cancer stem cell characteristics, and decreasing estrogen receptor (ER) expression [49]. Positive regulatory domain I (*PRDM1*) plays a critical role in TGF-β1-induced EMT and promotes breast cancer cell migration by inhibiting *BMP-5* expression [50]. E2F transcription factor 1 (*E2F1*) promotes TNBC proliferation and invasion by improving the expression of *CCNA2*, a key cell cycle regulator [51]. Yin Yang 1 (*YY1*) modulates the transcriptional activation of defective in cullin neddylation 1 domain containing 5 (*DCUN1D5*), which accelerates TNBC progression by targeting the *FN1/PI3K/AKT* signaling pathway [52]. Nuclear factor erythroid 2-related factor 1 (*NRF1*) upregulates proteasome gene expression to counteract proteasome inhibition in TNBC and maintain protein homeostasis for cancer cell survival [53]. Nuclear transcription factor Y subunit alpha (*NFYA*) promotes malignant behavior of TNBC in mice through the regulation of lipid metabolism. Overexpression of *NFYA* and its splice variants correlates with poor prognosis in breast cancer, making *NFYA* a significant therapeutic target and prognostic marker in TNBC [54]. Additionally, the miRNAs associated with the identified hub genes were determined, providing further insight into the regulatory network. hsa-miR-192-5p regulates key signaling pathways in TNBC cells by targeting genes involved in migration, such as *ARHGAP19*. Its overexpression inhibits proliferation, induces apoptosis, and suppresses migration. The tumor-suppressive impact is accomplished by epigenetic regulation and the direct interaction of miR-192 with *ARHGAP19* [55]. hsa-miR-186-5p is downregulated in TNBC cells, resulting in increased SBEM levels that activate the *PI3K/AKT* signaling pathway. This activation amplifies the migration, invasion, and proliferation of TNBC cells, underscoring miR-186-5p’s potential function as a tumor suppressor in TNBC progression [56]. The expression of miR-10b promotes metastasis and confers stem cell-like characteristics, such as chemoresistance, to breast cancer cells. Elevated miR-10b levels are associated with increased invasion, migration, and proliferation, underscoring its potential as a therapeutic target for aggressive breast cancer subtypes [57]. miR-92a-3p exhibits elevated expression in breast cancer tissues and cell lines, with greater levels correlating to increasing tumor size and advanced clinical stages [58]. The expression of miR-215-5p in breast cancer plays a crucial role in modulating tumor growth. It regulates circular RNAs, affecting key cellular activities and TNBC therapeutic response, potentially affecting patient prognosis and treatment outcomes [59]. The expression of miR-193b in TNBC correlates with reduced activity of key signaling pathways, including WNT/β-catenin and c-Met. It acts as a strong repressor of cell proliferation, migration, and stem-cell traits. Low miR-193b expression in TNBC patients correlates with increased aggressiveness and poor prognosis [60].

Repurposing existing drugs that aim at these hub genes could speed up the discovery of novel therapeutics for TNBC. The Drug–gene interaction analysis identified approved drugs that target the hub genes. Here, 29 drugs were associated with *TOP2A*, 6 with *CDK1*, and 3 with *CCNA2*. No drug interactions were identified for *BUB1, BUB1B, CCNB1, KIF20A, KIF11, MELK*, and *CENPF*. In DGIdb, interaction scores measure the strength and specificity of Drug–gene interaction, with higher scores indicating robust evidence. Genistein emerged as a common regulator of *TOP2A* and *CCNA2*. This compound inhibits protein-tyrosine kinase and topoisomerase-II activity and is considered a therapeutic option for cancers such as prostate, bone, colorectal, glioma, breast, and bladder cancer [61]. *CCNA2* is regulated by Tamoxifen, Genistein, and Ethinyl estradiol. Tamoxifen, an FDA-approved selective estrogen receptor modulators (SERMs), has been pivotal in ER + breast cancer treatment since the 1970s, significantly reducing recurrence across all stages and ages [62]. Ethinylestradiol, synthesized in the 1930s by modifying oestradiol, is the primary estrogen in oral contraceptives [63]. Similarly, *CDK1* is regulated by clofibrate and cinnarizine, which act as anti-cholesteremic and anti-allergic agents, respectively. For *TOP2A*, regulatory drugs include teniposide, dexrazoxane, amsacrine, etoposide, cinoxacin, etoposide phosphate, daunorubicin citrate, valrubicin, and pixantrone. Etoposide and teniposide, derived from mayapple epipodophyllotoxins, are used in chemotherapy for testicular tumors, lung cancer, and pediatric leukemia. Amsacrine inhibits *TOP2* by intercalating its acridine ring into DNA, stabilizing the cleavage complex to induce cytotoxicity. Pixantrone, an azaanthracenedione, treats non-Hodgkin B-cell lymphoma through DNA intercalation. Dexrazoxane is the only FDA-approved treatment for Doxorubicin-induced cardiotoxicity [64]. Cinoxacin addresses urinary tract infections caused by *Enterobacter* species. Daunorubicin, a non-specific anthracycline antibiotic, targets various cancers, including breast cancer. Finally, valrubicin, a semisynthetic doxorubicin analog, is utilized in bladder cancer treatment [65]. While *CDK1, CCNA2*, and *TOP2A* have approved drug candidates, our study highlights the necessity of repurposing drugs that regulate the other identified hub genes.

One of the notable heterogeneous characteristics of TNBC is the variation in HER2 expression levels, which can be evaluated in clinical settings. Based on pathological evaluations using immunohistochemistry (IHC) and fluorescence *in situ* hybridization (FISH), TNBC is classified into two distinct groups: HER2-negative/HER2-zero TNBC (HER2-neg TNBC; IHC 0) and HER2-low TNBC (HER2-low TNBC; IHC 1+, or IHC 2+ and FISH-negative). The clinical significance of HER2-low status in breast cancer is increasingly recognized, particularly in relation to immune signature-based subtypes. According to Hu et al., patients with HER2-low TNBC exhibit more malignant clinical behavior and aggressive tumor biology compared to those with the HER2-negative phenotype, suggesting that HER2 heterogeneity is an important factor to consider in the clinical management of TNBC patients [66]. Owing to the lack of well-established *in vitro* cell culture models for HER2-low TNBC and the absence of clinical metadata (such as IHC scores) in the GEO datasets used in this study, direct classification of HER2-low tumors was not feasible. Nevertheless, HER2 heterogeneity remains a significant non-negligible factor when considering immunotherapy-based treatment strategies, highlighting the need for accurate and standardized HER2 expression assessment in TNBC patients for future clinical settings.

Collectively, *KIF20A* identified in our study has been implicated in other breast cancer subtypes, but their role in TNBC remains largely unexplored. The mRNA and protein expression analysis also confirm that *KIF20A* is over expressed in breast cancer tissues. Further, the survival plot demonstrated that patients have a significantly poorer long-term survival rate with *KIF20A* overexpression, and the prospective regulatory elements reported may provide a new direction in therapeutic intervention. Considering the wealth of data, we hypothesize that exploring the mechanism of *KIF20A*’s role in TNBC and repositioning the drugs targeting the identified hub genes holds a promising avenue for precision medicine-driven therapeutic strategies in the near future.

## Supplementary Information



## Figures and Tables

**Table 1:** Description of dataset attributes and significant genes identified in each dataset.

Dataset	Series ID	Platform ID	Platform	Control	TNBC	Total DEGS	FDR <0.05 & log2FC ≥ ±1.0	Up	Down
Microarray	GSE36295	GPL6244	Affymetrix Human Gene 1.0 ST Array	5	11	33,297	1433	804	629
	GSE37751	GPL6244	Affymetrix Human Gene 1.0 ST Array	47	14	33,297	1078	425	653
	GSE61724	GPL6244	Affymetrix Human Gene 1.0 ST Array	4	16	33,297	1324	761	563
	GSE38959	GPL4133	Agilent-014850 Whole Human Genome Microarray 4 × 44K G4112F	13	30	45,015	4120	2795	1325
RNA-seq	GSE58135	GPL11154	Illumina HiSeq 2000	21	42	24,445	9186	6068	3118

**Table 2:** Hub genes identified using cytoHubba based on their network topological parameters.

S.No.	Gene	Gene Name	Betweenness	Degree	MCC	MNC	Radiality	Stress	Closeness
1	*CDK1*	Cyclin-dependent kinase 1	1149.32	53	4.18E+17	52	3.803	6060	70.166
2	*BUB1B*	*BUB1* Mitotic Checkpoint Serine/Threonine Kinase B	886.564	47	4.18E+17	46	3.729	4480	66.833
3	*CCNA2*	Cyclin A2	410.285	42	4.11E+17	42	3.664	3558	64.083
4	*BUB1*	*BUB1* Mitotic Checkpoint Serine/Threonine Kinase	428.095	45	4.18E+17	45	3.710	3328	65.833
5	*CCNB1*	Cyclin B1	302.671	43	4.18E+17	43	3.655	2620	64.250
6	*KIF20A*	Kinesin Family Member 20A	292.780	45	4.18E+17	45	3.636	2488	64.583
7	*CENPF*	Centromere Protein F	416.342	40	4.11E+17	39	3.627	2444	62.666
8	*TOP2A*	DNA Topoisomerase II Alpha	302.023	41	4.18E+17	40	3.627	2008	63.083
9	*KIF11*	Kinesin Family Member 11	210.327	45	4.18E+17	45	3.636	1918	64.583
10	*MELK*	Maternal Embryonic Leucine Zipper Kinase	208.594	33	3.98E+17	33	3.543	1492	58.833

**Table 3:** Correlation between hub genes and immune cells identified using TIMER 2.0 database.^a^

S. No.	Hub genes	Purity		CD8 T cell		CD4 T cell		B cell		Macrophage		Neutrophil		DC	
						
		Rho	p	Rho	p	Rho	p	Rho	p	Rho	p	Rho	p	Rho	p
1	*CDK1*	0.230	**2.02E-13**	−0.049	0.123	−0.016	0.619	0.100	**0.002**	−0.052	1.04E-01	2.73E-01	**2.03E-18**	5.20E-02	1.04E-01
2	*BUB1B*	0.181	**8.80E-09**	−0.004	0.911	−0.037	0.248	0.075	**0.018**	0.035	2.70E-01	3.43E-01	**7.46E-29**	5.80E-02	6.74E-02
3	*CCNA2*	0.178	**1.60E-08**	−0.045	0.154	−0.007	0.831	0.047	0.140	0.186	**3.77E-09**	3.82E-01	**7.11E-36**	1.02E-01	**1.35E-03**
4	*BUB1*	0.130	**4.02E-05**	0.013	0.693	0.089	**0.004**	0.075	**0.018**	0.007	8.26E-01	4.01E-01	**1.18E-39**	1.16E-01	**2.58E-04**
5	*CCNB1*	0.217	**4.46E-12**	−0.049	0.126	−0.057	0.073	0.094	**0.003**	−0.049	1.23E-01	2.35E-01	**6.37E-14**	1.30E-02	6.73E-01
6	*KIF20A*	0.180	**1.18E-08**	0.001	0.973	−0.020	0.535	0.069	**0.031**	−0.018	5.78E-01	3.28E-01	**2.39E-26**	7.10E-02	**2.48E-02**
7	*CENPF*	0.165	**1.64E-07**	0.041	0.196	0.010	0.758	0.031	0.323	0.059	6.31E-02	3.55E-01	**6.72E-31**	6.70E-02	**3.59E-02**
8	*TOP2A*	0.246	**3.12E-15**	0.019	0.540	−0.052	0.102	0.023	0.467	0.080	**1.19E-02**	3.07E-01	**3.38E-23**	−1.00E-03	9.75E-01
9	*KIF11*	0.218	**3.45E-12**	0.011	0.721	−0.003	0.913	0.060	0.060	−0.020	5.35E-01	3.10E-01	**1.27E-23**	3.10E-02	3.31E-01
10	*MELK*	0.166	**1.45E-07**	−0.030	0.349	0.041	0.191	0.085	**0.008**	−0.075	**1.76E-02**	3.49E-01	**6.59E-30**	1.29E-01	**4.40E-05**

a Bold numbers indicate the significant values.

**Table 4:** Interaction scores highlighting the relationship between the hub gene and the drug.

S.No.	Gene	Drug	Interaction Score
1	*CCNA2*	Tamoxifen	0.276
2	*CCNA2*	Genistein	0.324
3	*CCNA2*	Ethinyl estradiol	0.991
4	*CDK1*	Cinnarizine	0.214
5	*CDK1*	Clofibrate	0.157
6	*TOP2A*	Enoxacin	0.263
7	*TOP2A*	Teniposide	0.351
8	*TOP2A*	Dexrazoxane	0.421
9	*TOP2A*	Amsacrine	0.456
10	*TOP2A*	Etoposide	0.490
11	*TOP2A*	Cinoxacin	0.527
12	*TOP2A*	Etoposide phosphate	0.527
13	*TOP2A*	Daunorubicin citrate	0.527
14	*TOP2A*	Valrubicin	1.053
15	*TOP2A*	Pixantrone	1.053

## Data Availability

All data analysed during this study are publicly available and are included in this article (and its Supplementary Information files).
