# Supplementary material for: Unveiling immune-related gene signatures in triple negative breast cancer through integrated transcriptomic analysis
Source: Biomedicine (Taipei). 2026 Jun 1;16(2):52–67. doi: 10.37796/2211-8039.1708 (PMC13387401; doi:10.37796/2211-8039.1708)
Supplement: Supplementary file 1 [file bmed-16-02-052-s001.docx]

**Unveiling immune-related gene signatures in triple negative breast cancer through integrated transcriptomic analysis**

Priyanga Paranthaman, Ramanathan Karuppasamy and Shanthi Veerappapillai^*^

Department of Biotechnology, School of Bio Sciences and Technology,

Vellore Institute of Technology, Vellore, Tamil Nadu, India.

*Corresponding author. Email: shanthi.v@vit.ac.in; Phone: 0416-2202625

**List of Figures**

**Fig.** **S1** Box plot of (**a**) *CDK1* (**b**) *BUB1B* (**c**) *CCNA2* (**d**) *BUB1* (**e**) *CCNB1* (**f**) *KIF20A* (**g**) *CENPF* (**h**) *TOP2A* (**i**) *KIF11* (**j**) *MELK*. mRNA level in BRCA dataset (Red) with matched TCGA normal and GTEx data (Grey). (*) red asterisk indicates a p-value <0.05; BRCA - Breast invasive carcinoma.

**Fig. S2** Correlation between the expression of hub genes and tumor purity along with infiltrating levels of CD8^+^ T cells, CD4^+^ T cells, B cells, macrophages, neutrophils, and dendritic cells in Breast Invasive Carcinoma (BRCA). (**a**) *CDK1* (**b**) *BUB1B* (**c**) *CCNA2* (**d**) *BUB1* (**e**) *CCNB1* (**f**) *KIF20A* (**g**) *CENPF* (**h**) *TOP2A* (**i**) *KIF11* (**j**) *MELK.*

**Fig.** **S3** Overall survival (OS) analysis of ten hub genes in BRCA dataset (**a**) *CDK1* (**b**) *BUB1B* (**c**) *CCNA2* (**d**) *BUB1*(**e**) *CCNB1*(**f**) *KIF20A* (**g**) *CENPF* (**h**) *TOP2A* (**i**) *KIF11* (**j**) *MELK*.

| **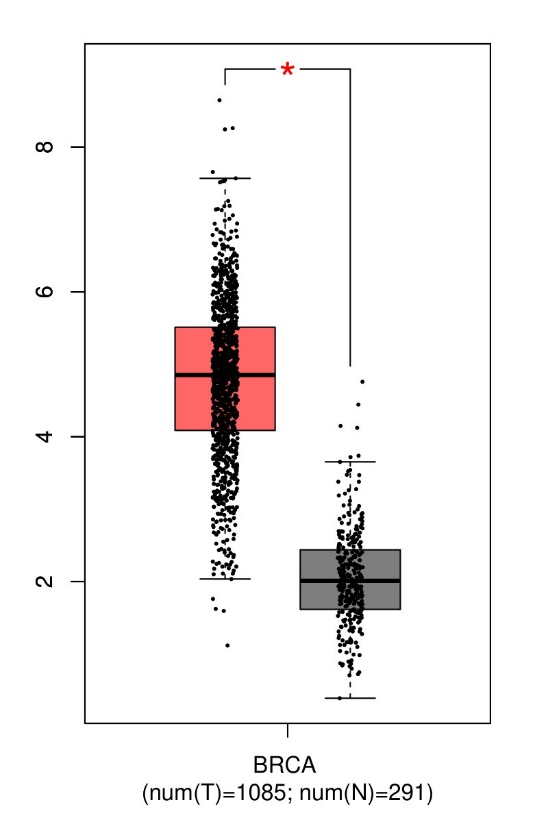** | **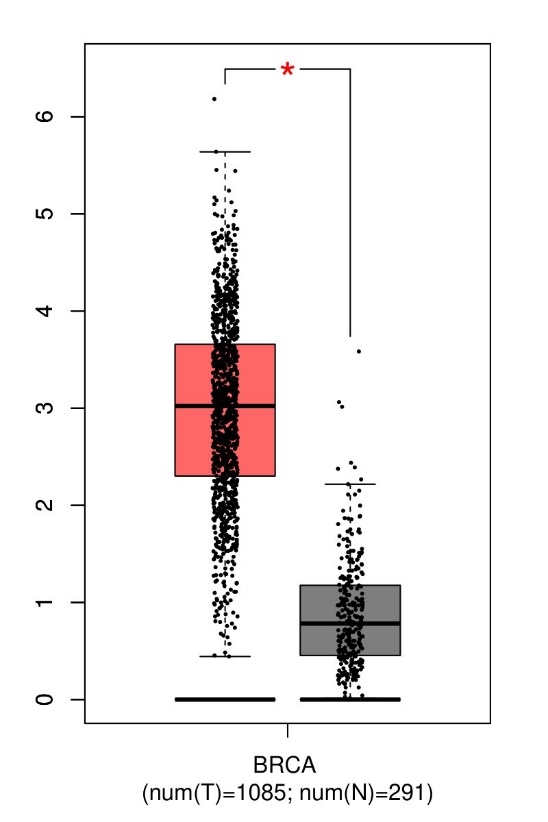** |
| --- | --- |
| **(a)** | **(b)** |
| **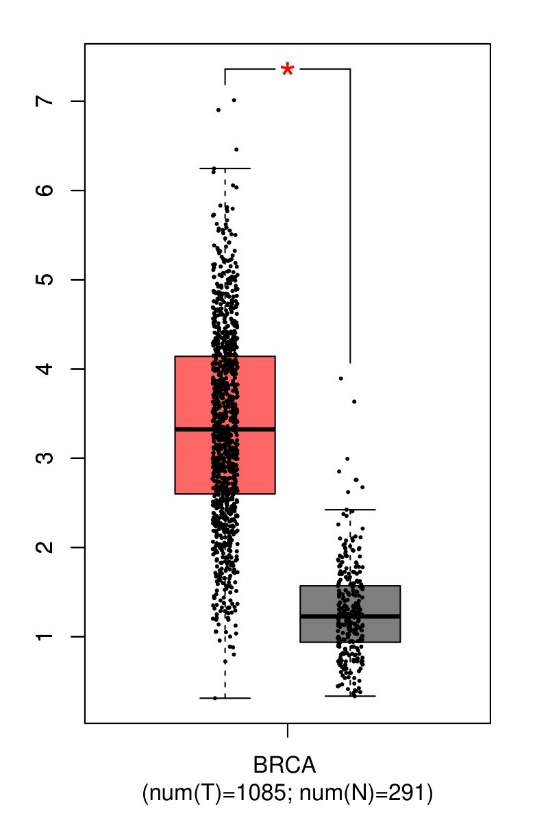** | **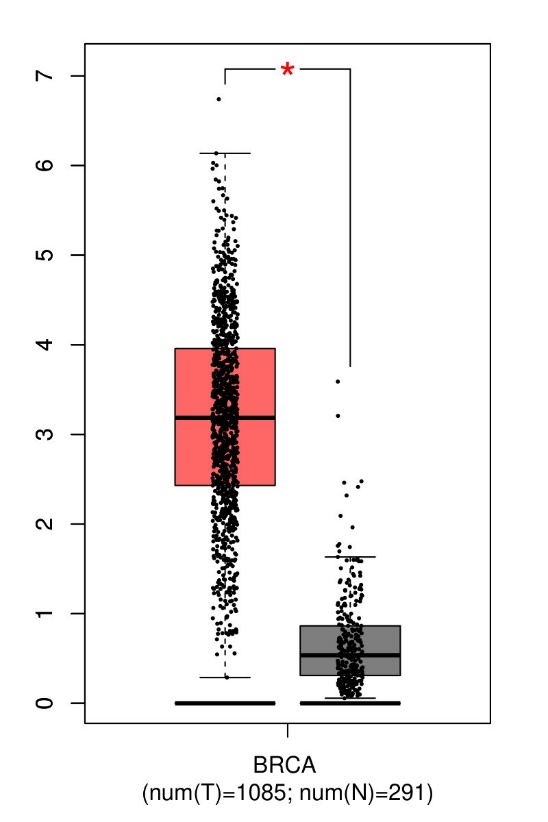** |
| **(c)** | **(d)** |
| **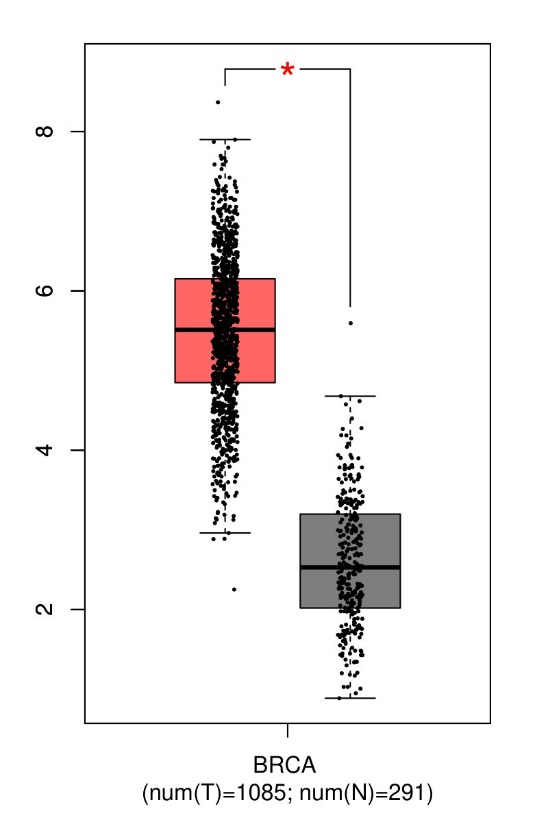** | **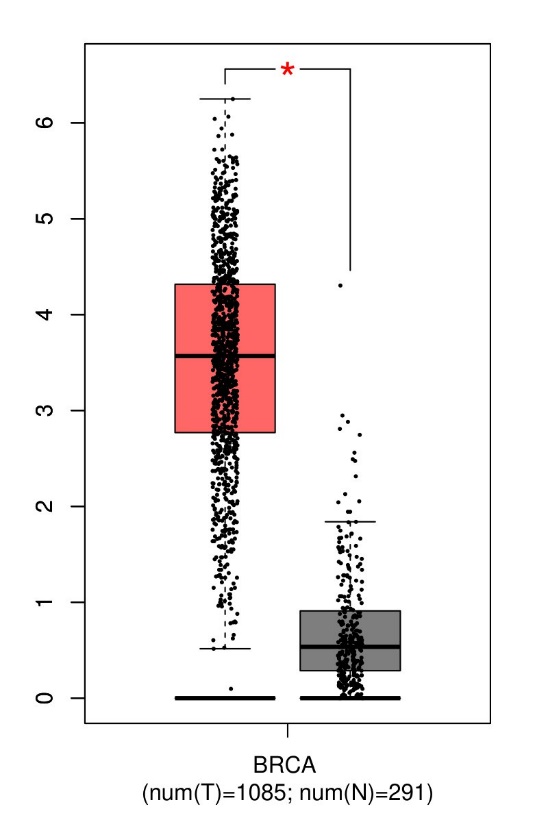** |
| **(e)** | **(f)** |
| **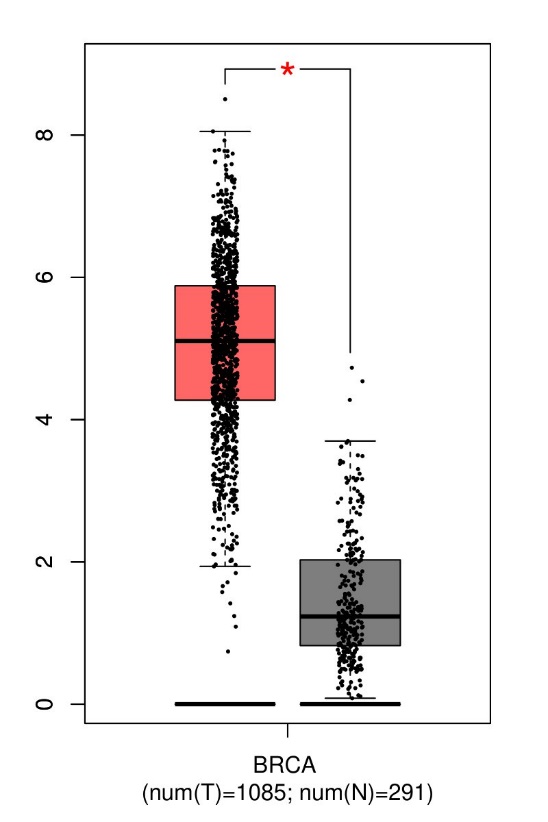** | **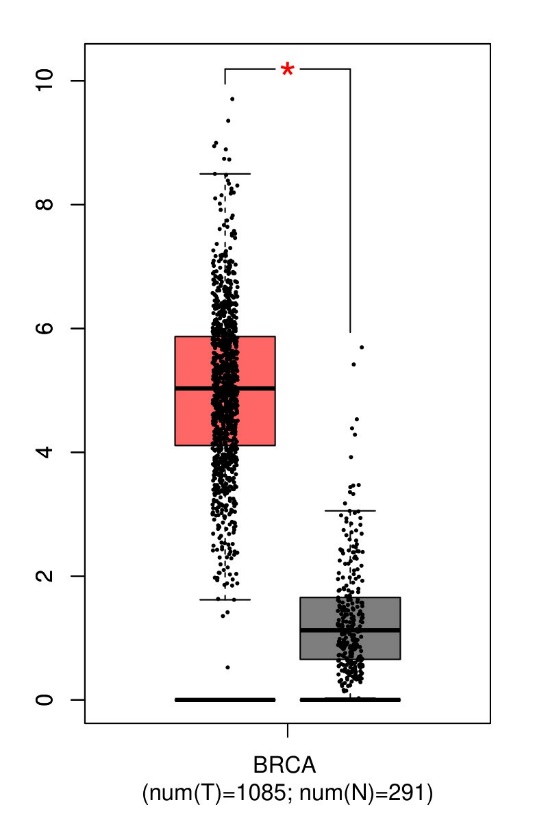** |
| **(g)** | **(h)** |
| **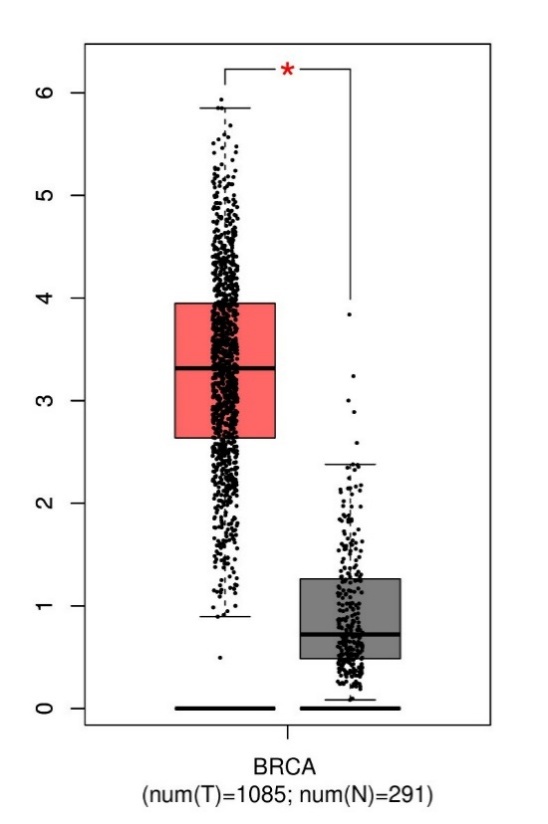** | **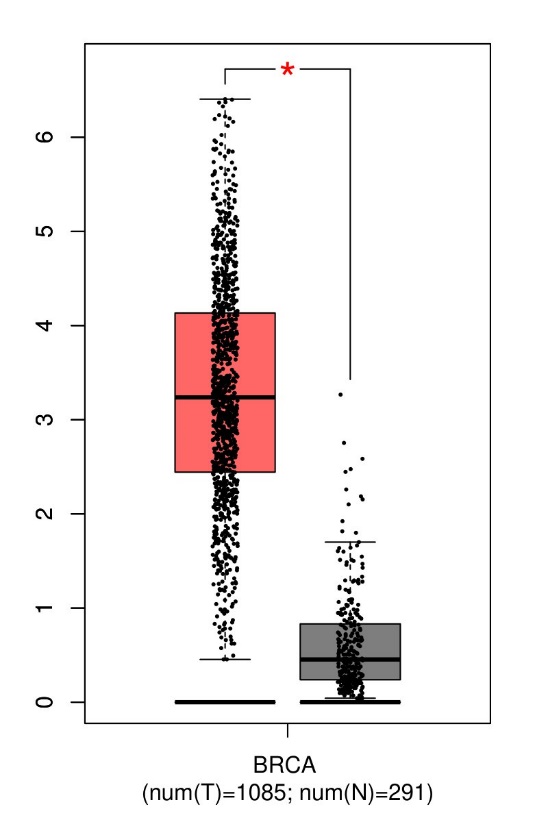** |
| **(i)** | **(j)** |
| **Fig. S1** Box plot of (**a**) *CDK1* (**b**) *BUB1B* (**c**) *CCNA2* (**d**) *BUB1* (**e**) *CCNB1* (**f**) *KIF20A* (**g**) *CENPF* (**h**) *TOP2A* (**i**) *KIF11* (**j**) *MELK*. mRNA level in BRCA dataset (Red) with matched TCGA normal and GTEx data (Grey). (*) red asterisk indicates a p-value <0.05; BRCA - Breast invasive carcinoma. | |

| 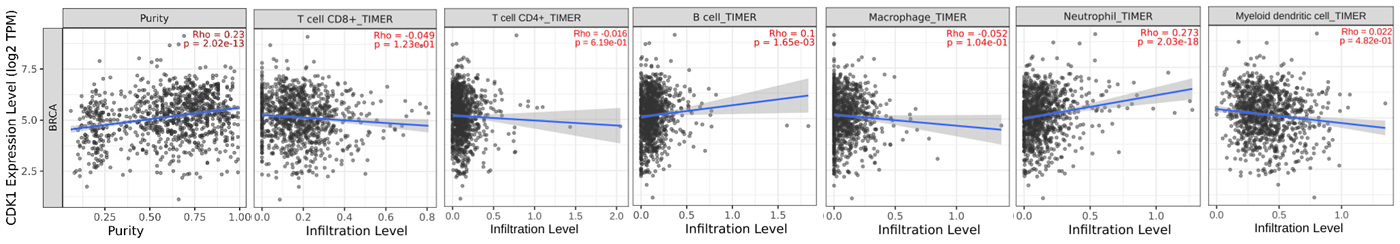 |
| --- |
| **(a)** |
| 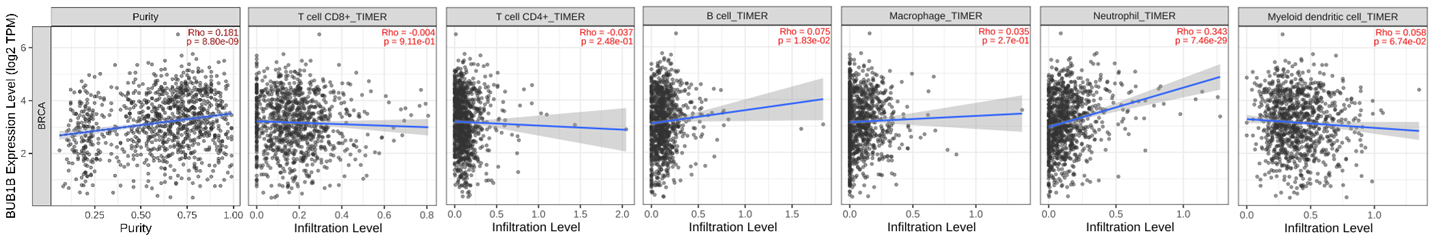 |
| **(b)** |
| 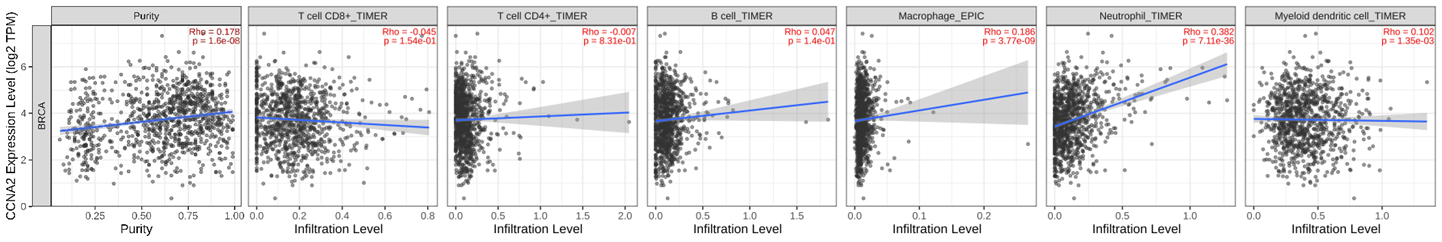 |
| **(c)** |
| 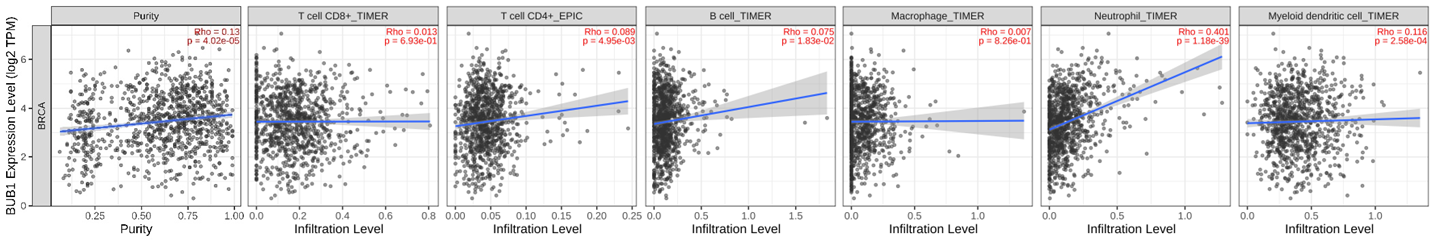 |
| **(d)** |
| 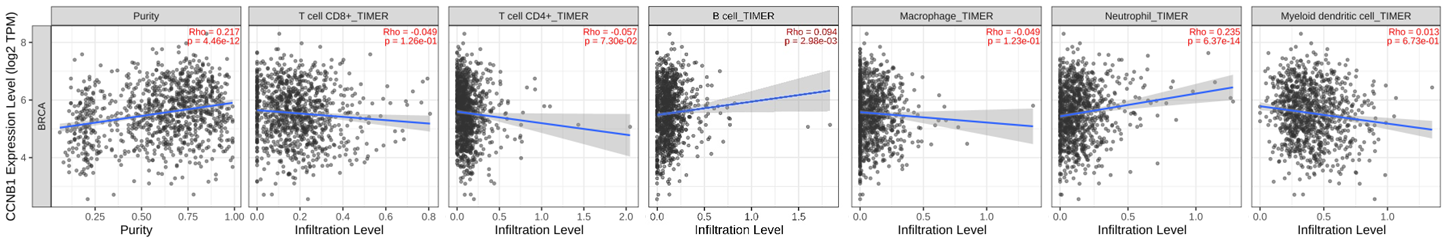 |
| **(e)** |
| 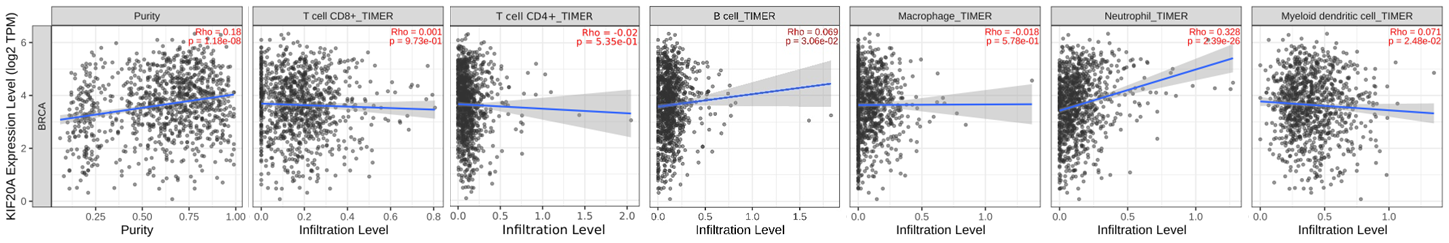 |
| **(f)** |
| 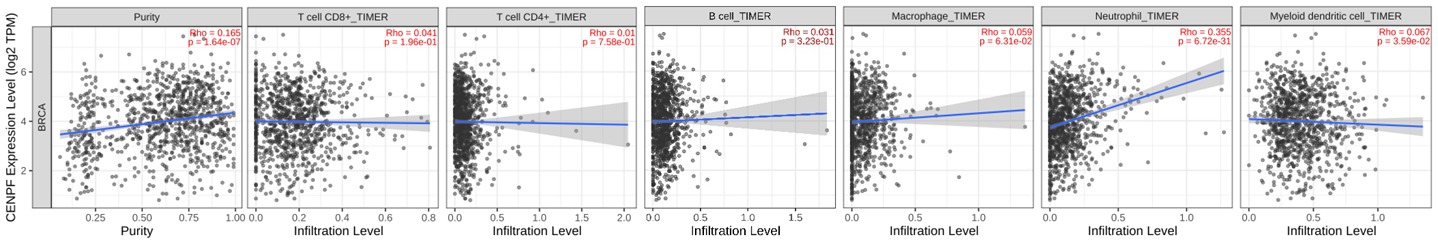 |
| **(g)** |
| 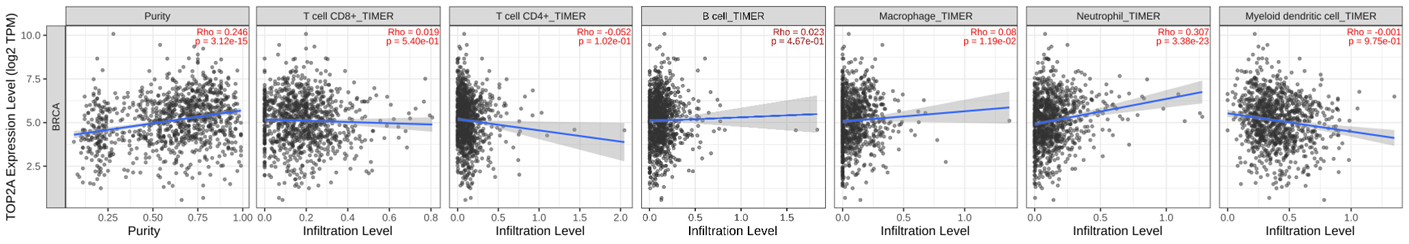 |
| **(h)** |
| 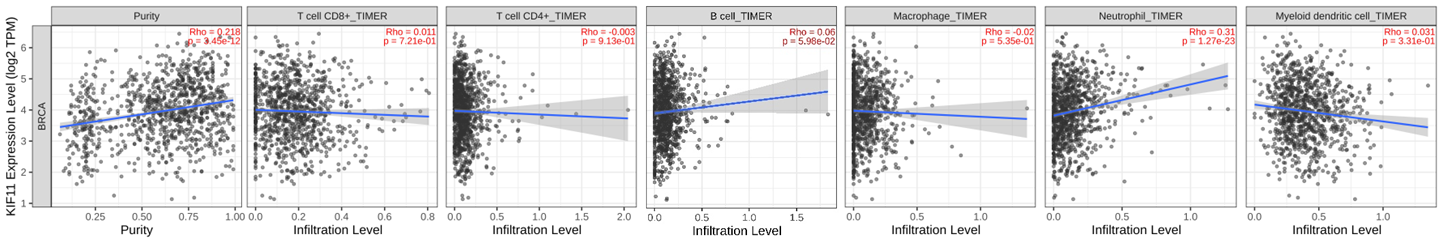 |
| **(i)** |
| 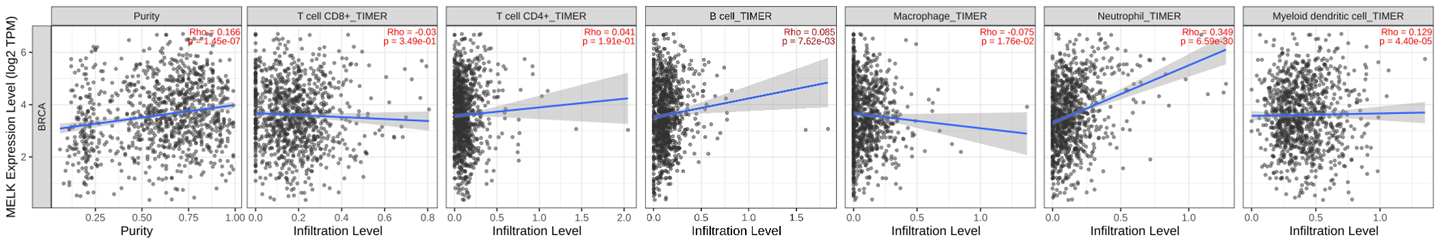 |
| **(j)** |
| **Fig. S2** Correlation between the expression of hub genes and tumor purity along with infiltrating levels of CD8^+^ T cells, CD4^+^ T cells, B cells, macrophages, neutrophils, and dendritic cells in Breast Invasive Carcinoma (BRCA). (**a**) *CDK1* (**b**) *BUB1B* (**c**) *CCNA2* (**d**) *BUB1* (**e**) *CCNB1* (**f**) *KIF20A* (**g**) *CENPF* (**h**) *TOP2A* (**i**) *KIF11* (**j**) *MELK.* |
|  |

| **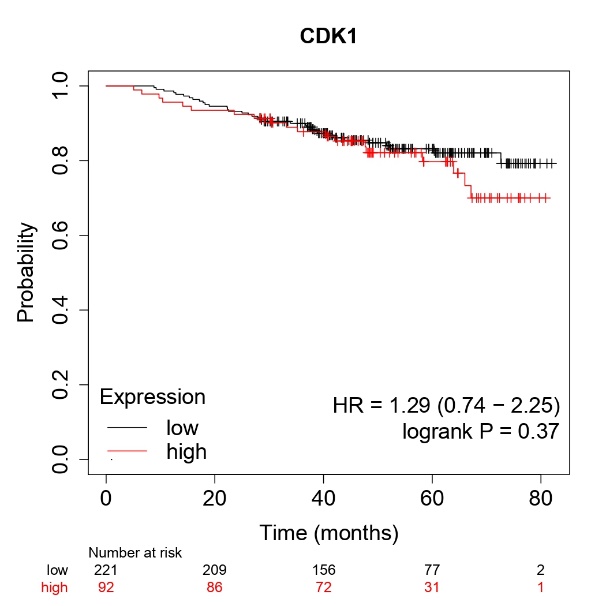** | **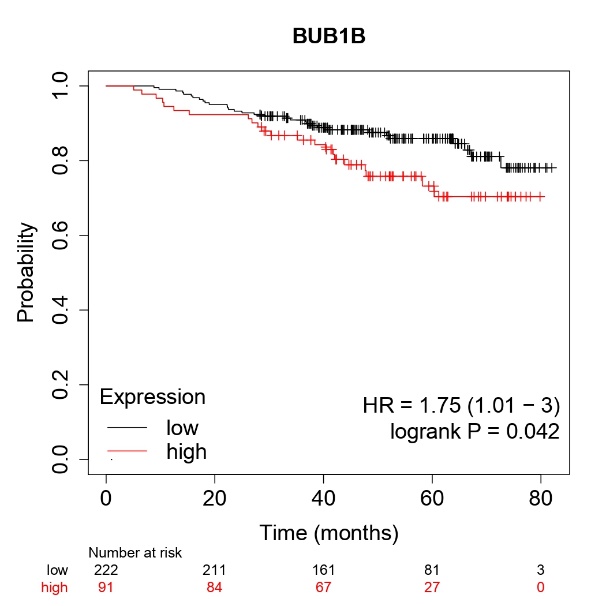** |
| --- | --- |
| **(a)** | **(b)** |
| **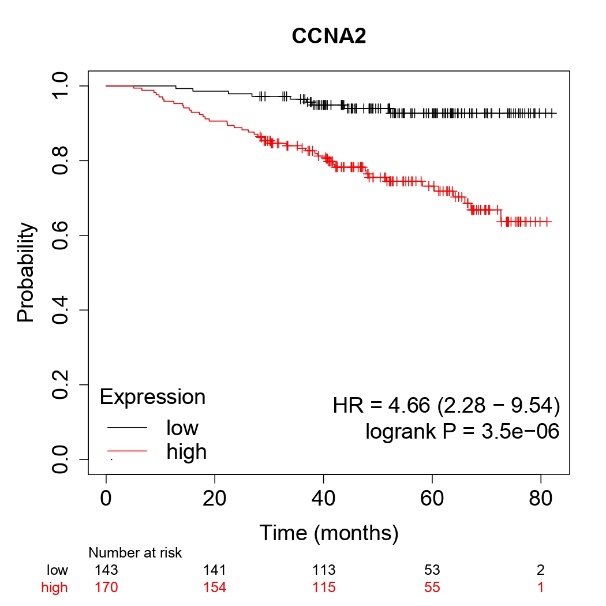** | **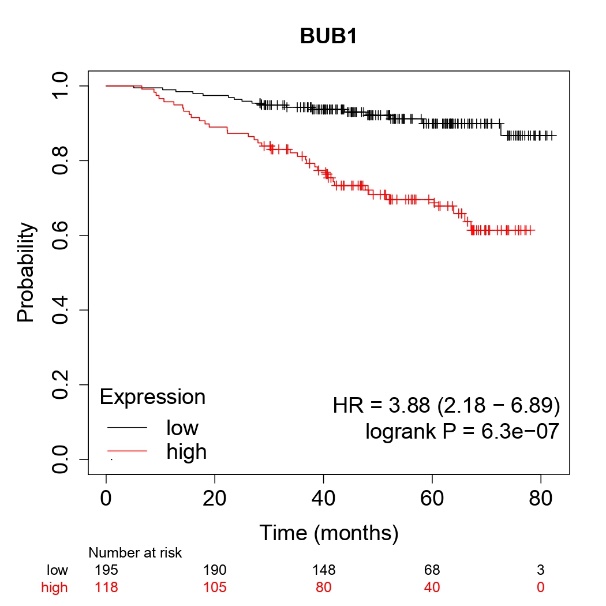** |
| **(c)** | **(d)** |
| **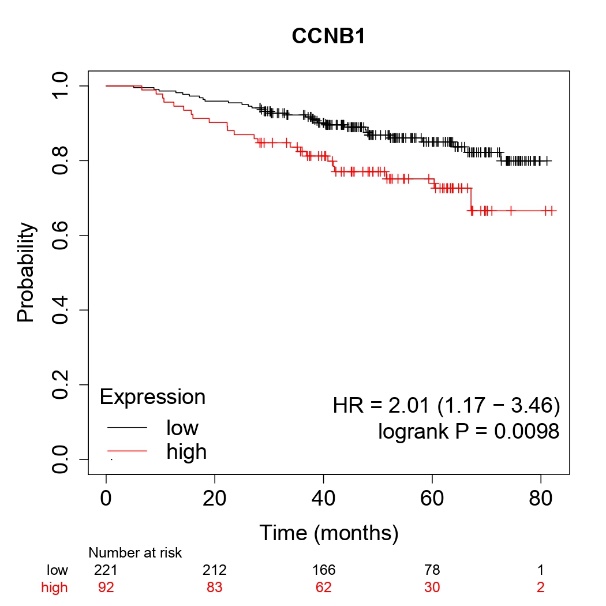** | **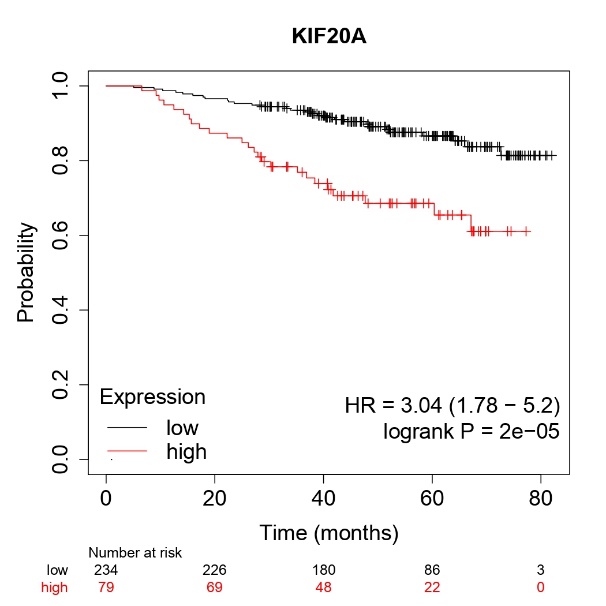** |
| **(e)** | **(f)** |
| **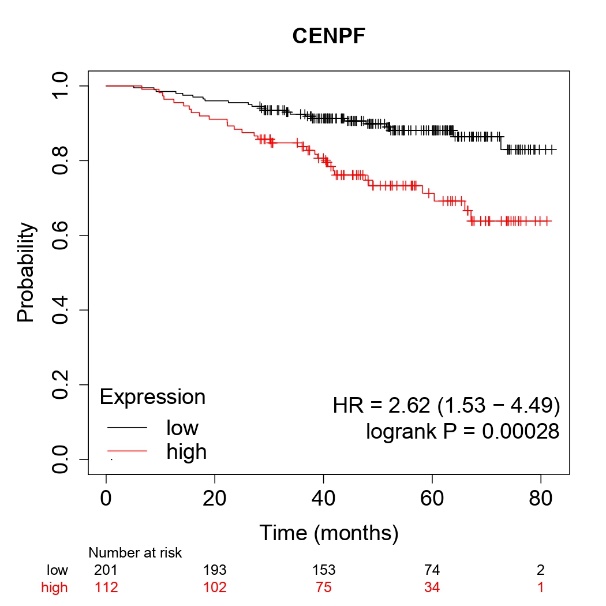** | **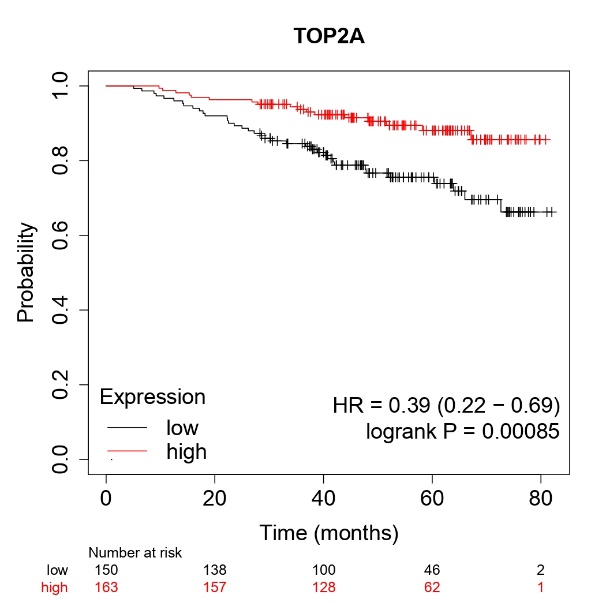** |
| **(g)** | **(h)** |
| **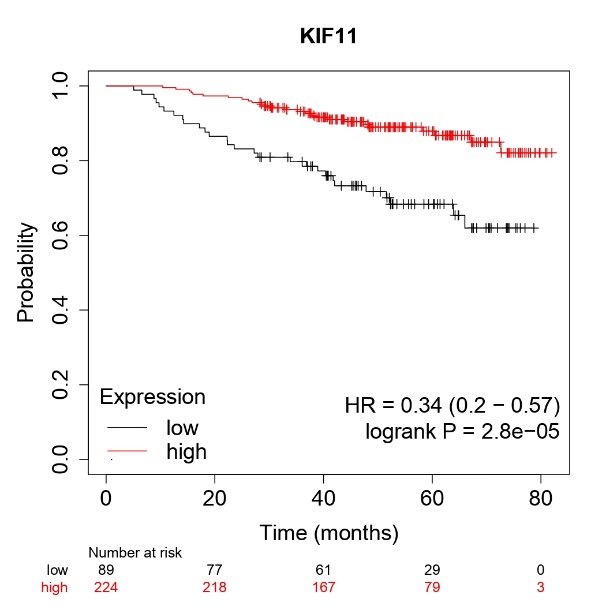** | **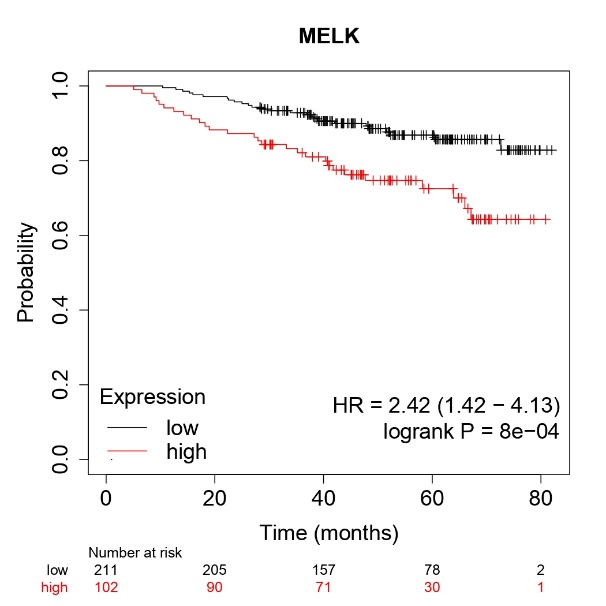** |
| **(i)** | **(j)** |
| **Fig. S3** Overall survival (OS) analysis of ten hub genes in BRCA dataset. (**a**) *CDK1* (**b**) *BUB1B* (**c**) *CCNA2* (**d**) *BUB1* (**e**) *CCNB1* (**f**) *KIF20A* (**g**) *CENPF* (**h**) *TOP2A* (**i**) *KIF11* (**j**) *MELK.* | |
